# Validation of data mining models by comparing with conventional methods for dental age estimation in Korean juveniles and young adults

**DOI:** 10.1038/s41598-023-28086-1

**Published:** 2023-01-13

**Authors:** Akiko Kumagai, Seoi Jeong, Daeyoun Kim, Hyoun-Joong Kong, Sehyun Oh, Sang-Seob Lee

**Affiliations:** 1 Division of Forensic Odontology and Disaster Oral Medicine, Department of Forensic Science, Iwate Medical University, Iwate, 028-3694 Japan; 2 Interdisciplinary Program in Bioengineering, Graduate School, Seoul National University, Seoul, 03080 Republic of Korea; 3 Kakao Corp., Jeju, 63309 Republic of Korea; 4 Transdisciplinary Department of Medicine and Advanced Technology, Seoul National University Hospital, Seoul, 03080 Republic of Korea; 5 Medical Big Data Research Center, Seoul National University College of Medicine, Seoul, 03080 Republic of Korea; 6 Department of Biomedical Engineering, Seoul National University College of Medicine, Seoul, 03080 Republic of Korea; 7 Department of Anatomy, Catholic Institute of Applied Anatomy, College of Medicine, The Catholic University of Korea, Seoul, 06591 Republic of Korea

**Keywords:** Dental anthropology, Forensic dentistry, Oral anatomy

## Abstract

Teeth are known to be the most accurate age indicators of human body and are frequently applied in forensic age estimation. We aimed to validate data mining-based dental age estimation, by comparing the accuracy of the estimation and classification performance of 18-year thresholds with conventional methods and with data mining-based age estimation. A total of 2657 panoramic radiographs were collected from Koreans and Japanese populations aged 15 to 23 years. They were subdivided into a training and internal test set of 900 radiographs each from Koreans, and an external test set of 857 radiographs from Japanese. We compared the accuracy and classification performance of the test sets from conventional methods with those from the data mining models. The accuracy of the conventional method with the internal test set was slightly higher than that of the data mining models, with a slight difference (mean absolute error < 0.21 years, root mean square error < 0.24 years). The classification performance of the 18-year threshold was also similar between the conventional method and the data mining models. Thus, conventional methods can be replaced by data mining models in forensic age estimation using second and third molar maturity of Korean juveniles and young adults.

## Introduction

Dental age estimation is widely used in forensic science and pediatric dentistry. In particular, age estimation using the developmental stages of teeth is an important criterion for estimating the age of children and adolescents, due to the high correlation between chronological age and teeth development^1–3^. However, in the case of young adults, dental age estimation with teeth maturity has limitations because the growth of teeth is largely complete, except the third molars. The legal purpose of age estimation for young adults and adolescents is to provide an accurate estimation and scientific evidence as to whether they have reached the age of majority. In Korean forensic practice for juveniles and young adults, age was estimated with Lee’s method^4^, and the legal 18-year threshold was predicted with the data suggested by Oh et al.^5^.

Machine learning, a type of artificial intelligence (AI), repeatedly learns and categorizes large amounts of data, solves problems on its own, and induces data programming. Machine learning can discover useful hidden patterns within a large amount of data^6^. In contrast, labor-intensive and time-consuming classical methods may have limitations in dealing with large amounts of complex data that are difficult to process manually^7^. Thus, many studies have been conducted recently using the latest computer technology to minimize human error and efficiently process multi-dimensional data^8–12^. In particular, deep learning is widely used for medical image analysis, and various methods have been reported to estimate age by automatically analyzing radiographs to improve the accuracy and efficiency of age estimation^13–20^. For example, Halabi et al.^13^ developed machine learning algorithms based on convolutional neural networks (CNN) using pediatric hand radiographs to estimate the age of bones. This study presented a model that applied machine learning to medical images and showed that these techniques can aid diagnostic accuracy. Li et al.^14^ estimated age from X-ray images of pelvic bones by applying deep learning CNN and compared with the results of regression analysis using the evaluation of the ossification stages. They found that the deep learning CNN model showed the same age estimation performance as the conventional regression model. A study by Guo et al.^15^ evaluated the classification performance of legal age thresholds by applying CNN technology based on dental orthopantomograms, and the result of the CNN model proved that humans outperformed its age classification performance.

Most of the age estimation studies performed with machine learning use deep learning methods^13–20^. Deep learning-based age estimation has been reported to be more accurate than conventional methods. However, this method makes it almost impossible to present the scientific basis of age estimation, such as the age indicators used in the estimation. In addition, there was a legal controversy about who performed the examination. Therefore, deep learning-based age estimation is difficult to accept by administrative and judicial agencies. Data mining (DM) is a technique that can find not only expected but also unexpected information as a method to discover useful correlations among a large amount of data^6,21,22^. When conducting DM, machine learning is usually used and both DM and machine learning employ the same critical algorithms to discover data patterns. Age estimation using tooth development is based on the examiner's maturity evaluation of the targeted teeth, and this evaluation is expressed as the stage of each targeted tooth. DM can be used to analyze the correlation between the evaluated stages of the teeth and their chronological age and has the potential to replace conventional statistical analysis. Therefore, if we apply DM approaches to age estimation, we can introduce machine learning to forensic age estimation free of legal responsibility concerns. Some comparative studies on the possible substitution of conventional manual methods used in forensic practice with the DM-based methods in dental age estimation have been published. Shen et al.^23^ showed that the DM models were more accurate than the traditional Cameriere formula. Galibourg et al.^24^, who predicted age by applying various DM methods based on Demirjian’s criteria^25^, showed that the DM methods were superior to the Demirjian’s and Willems methods in age estimation for the French population.

For the dental age estimation of Korean juveniles and young adults, Lee’s method^4^ has been widely used in Korean forensic practice. This method uses conventional statistical analysis, such as multiple regression, to examine the relationship between Korean subjects and chronological ages. In this study, the age estimation method derived through conventional statistical techniques was defined as a "conventional method." The accuracy of Lee's method, which is a conventional method, has already been validated by Oh et al.^5^; however, the applicability of age estimation based on DM models in Korean forensic practice remains questionable. We aimed to scientifically validate the potential utility of age estimation based on DM models. The objectives of this study were to (1) compare the accuracy of two DM models in dental age estimation and (2) compare the classification performance of the 18-year threshold of seven DM models and the methods derived from conventional statistical approaches using the maturity of the second and third molars in both jaws.

## Results

### Observer reliability

The means and standard deviations of chronological ages according to stages and types of teeth are presented in Supplementary Table S1 (Training set), Supplementary Table S2 (Internal test set), and Supplementary Table S3 (External test set) online. The kappa values, of the intra- and inter-observer reliability obtained in the training set were 0.951 and 0.947, respectively. The p-values and 95% confidence intervals of kappa values are presented in Supplementary Table S4 online. The kappa values were construed as “almost perfect,” consistent with Landis and Koch’s standards^26^.

### Accuracy of age estimation

**Figure 1:**
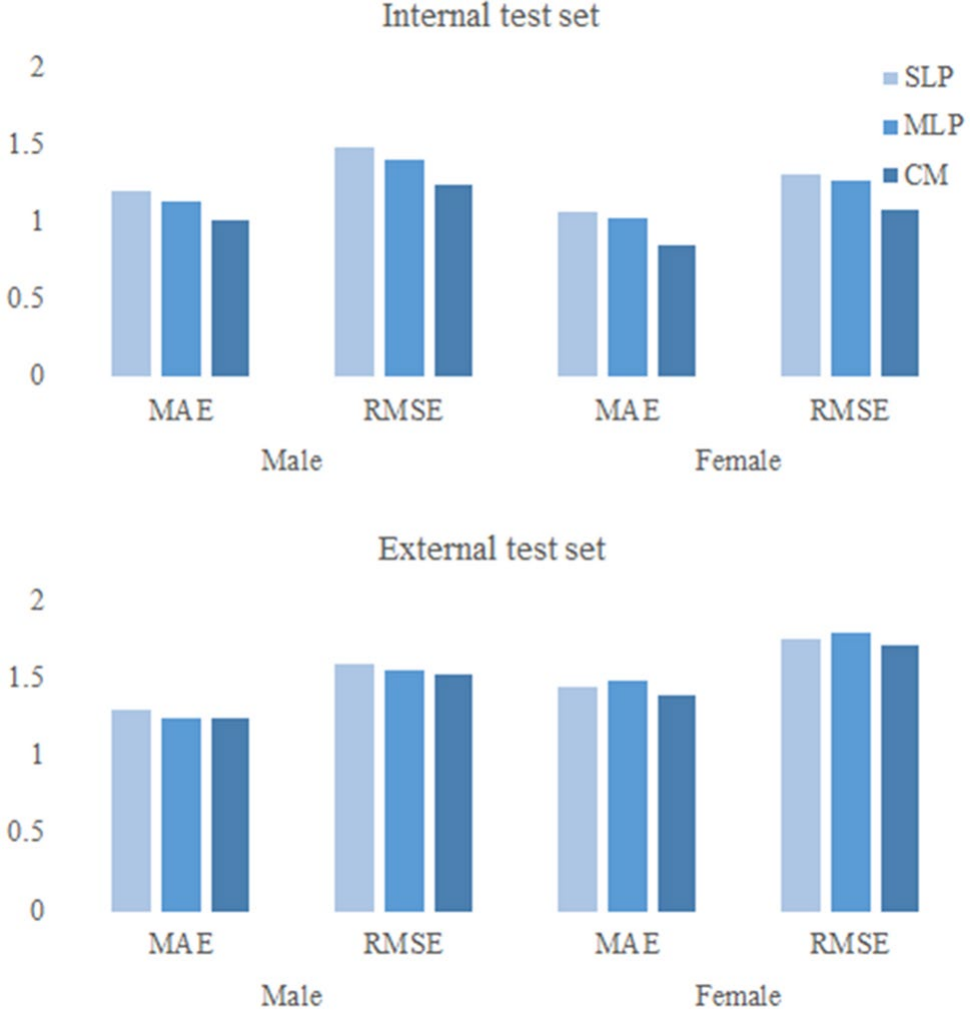
MAE and RMSE of conventional regression and data mining regression models. *MAE* mean absolute error, *RMSE* root mean square error, *SLP* single layer perceptron, *MLP* multilayer perceptron, *CM* conventional method.

When comparing the mean absolute error (MAE), the conventional methods were marginally better than the DM models in all sexes and test sets other than the multilayer perceptron (MLP) in the male external test set. The differences between the conventional and DM models for the internal test sets in MAE were 0.12 –0.19 years in males and 0.17 ~ 0.21 years in females. For the external test sets, the differences were smaller (0.001 – 0.05 years in males, 0.05 –0.09 years in females). In addition, the root mean square error (RMSE) had slightly lower values with the conventional method with small differences (0.17– 0.24, 0.2 –0.24 for the internal test sets, 0.03 ~ 0.07, 0.04 ~ 0.08 for the external test sets in males and females, respectively). Other than the case of the female external test set, the MLP showed slightly better performance than the single layer perceptron (SLP). With both MAE and RMSE, the external test set results were higher than those of the internal test sets in all sexes and models. All the MAE and RMSE are shown in Table 1 and Fig. 1.

**Table 1:** MAE and RMSE of conventional regression and data mining regression models.

Internal test set	Male		Female	
Method	MAE	RMSE	MAE	RMSE
Single layer perceptron	1.1974	1.4814	1.0634	1.3165
Multilayer perceptron	1.1325	1.4125	1.0308	1.2776
Conventional method	1.0155	1.2398	0.8539	1.0772

External test set	Male		Female	
Method	MAE	RMSE	MAE	RMSE
Single layer perceptron	1.3011	1.5957	1.4500	1.7611
Multilayer perceptron	1.2487	1.5513	1.4877	1.8025
Conventional method	1.2497	1.5241	1.3996	1.7168

*MAE* mean absolute error, *RMSE* root mean square error.

### Classification of the 18-year threshold

**Figure 2:**
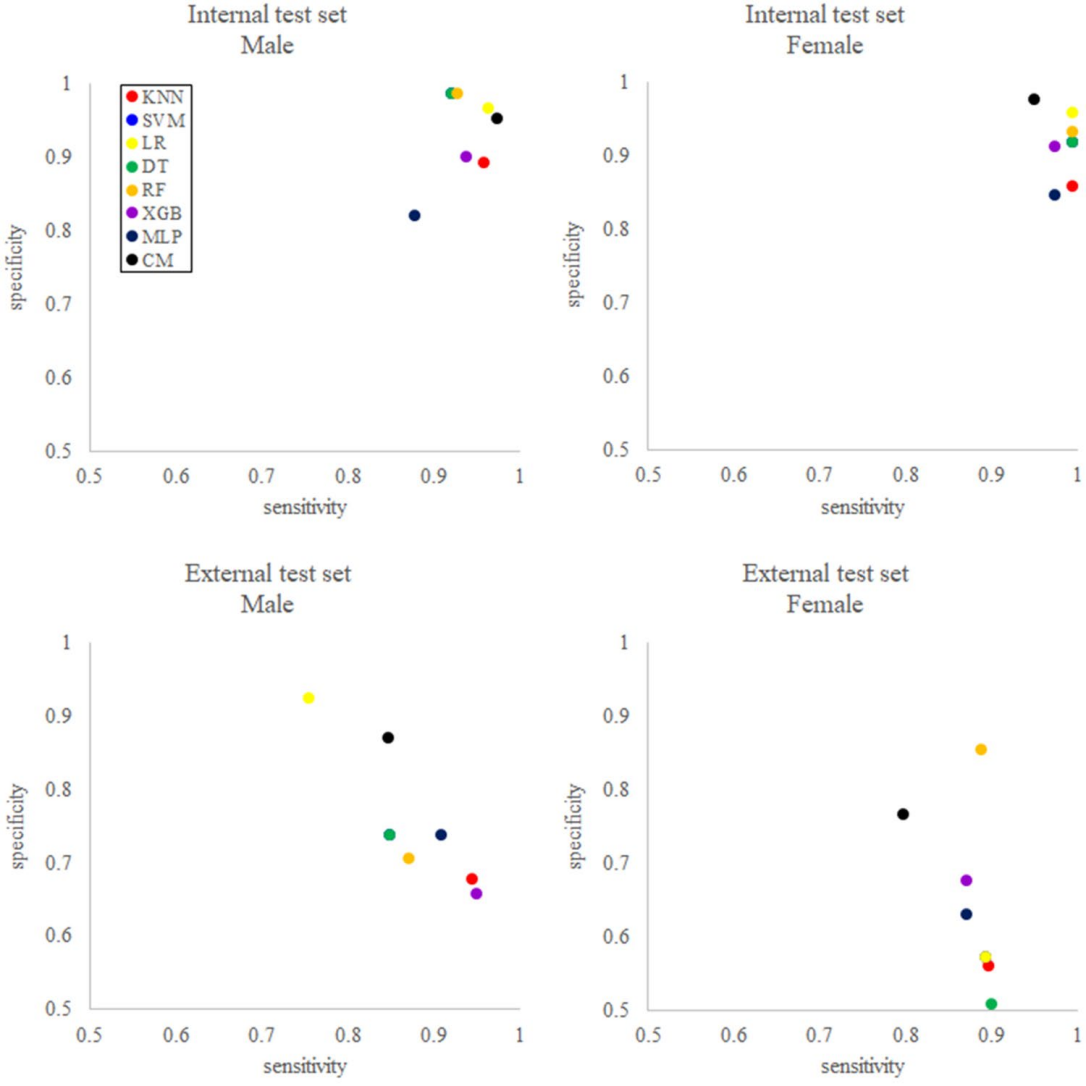
Sensitivity and specificity of the data mining classification models and the conventional method, with 18-year threshold. *KNN k*-nearest neighbor, *SVM* support vector machine, *LR* logistic regression, *DT* decision tree, *RF* random forest, *XGB* XGBoost, *MLP* multilayer perceptron, *CM* conventional method.

The classification performance of both the conventional and DM models, with the 18-year threshold, was demonstrated as sensitivity, specificity, positive predictive value (PPV), negative predictive value (NPV), and area under the receiver operating characteristic curve (AUROC)^27^ (Table 2, Fig. 2, and Supplementary Fig. 1 online). For the sensitivity of the internal test set, the conventional method demonstrated the best performance in males and the worst performance in females. However, the difference in classification performance between the conventional method and DM was 9.7% (MLP) in males, while in females, it was only 2.4% (XGBoost). Among the DM models, logistic regression (LR) showed the best sensitivity performance in both sexes. Regarding the specificity of the internal test set, four DM models were observed to be superior in males, while the conventional models demonstrated the best performance in females. The differences in classification performance were 13.3% (MLP) and 13.1% (MLP) in males and females, respectively, indicating that the difference in classification performance between the models was larger than the sensitivity. Among the DM models, the performance of the support vector machine (SVM), decision tree (DT), and random forest (RF) models were best in males, and LR was best in females. The AUROC of the conventional and all DM models was greater than 0.925 (k-nearest neighbor (KNN) in males) demonstrating excellent classification performance in discriminating 18-year samples^28^. In the case of the external test set, a decline in the classification performance was observed in sensitivity, specificity, and AUROC compared to those of the internal test set. Additionally, the difference between the classification performance of the best and worst models, in both sensitivity and specificity, was 10 to 25%, and larger than that in the internal test set.

**Table 2:** The classification performance of the data mining classification models and the conventional method based on the 18-year threshold.

Internal test set	Method	Sensitivity	Specificity	PPV	NPV	AUROC
Male	KNN	0.957	0.893	0.947	0.912	0.925
	SVM	0.920	0.987	0.993	0.860	0.989
	Logistic regression	0.963	0.967	0.983	0.929	0.988
	Decision tree	0.920	0.987	0.993	0.860	0.976
	Random forest	0.927	0.987	0.993	0.873	0.984
	XGBoost	0.937	0.900	0.949	0.877	0.970
	MLP	0.877	0.820	0.907	0.769	0.939
	Conventional method	0.974	0.953	0.977	0.947	0.990

Internal test set	Method	Sensitivity	Specificity	PPV	NPV	AUROC
Female	KNN	0.993	0.860	0.934	0.985	0.949
	SVM	0.993	0.920	0.961	0.986	0.969
	Logistic regression	0.993	0.960	0.980	0.986	0.982
	Decision tree	0.993	0.919	0.958	0.986	0.967
	Random forest	0.993	0.933	0.968	0.986	0.973
	XGBoost	0.973	0.913	0.957	0.945	0.953
	MLP	0.987	0.847	0.928	0.969	0.940
	Conventional method	0.949	0.978	0.990	0.893	0.998

External test set	Method	Sensitivity	Specificity	PPV	NPV	AUROC
Male	KNN	0.945	0.679	0.857	0.858	0.857
	SVM	0.849	0.739	0.868	0.707	0.813
	Logistic regression	0.754	0.925	0.953	0.649	0.810
	Decision tree	0.849	0.739	0.868	0.707	0.813
	Random forest	0.871	0.707	0.856	0.729	0.815
	XGBoost	0.949	0.657	0.849	0.863	0.852
	MLP	0.908	0.739	0.876	0.798	0.852
	Conventional method	0.846	0.870	0.952	0.649	0.912

External test set	Method	Sensitivity	Specificity	PPV	NPV	AUROC
Female	KNN	0.896	0.561	0.766	0.770	0.767
	SVM	0.892	0.572	0.770	0.767	0.769
	Logistic Regression	0.892	0.572	0.770	0.767	0.769
	Decision Tree	0.899	0.509	0.746	0.759	0.749
	Random Forest	0.888	0.854	0.774	0.765	0.772
	XGBoost	0.871	0.676	0.812	0.765	0.796
	MLP	0.871	0.630	0.791	0.752	0.778
	Conventional Method	0.797	0.766	0.878	0.642	0.873

The 95% CI of sensitivity, specificity, PPV, NPV, and AUROC calculated by the conventional method are presented in Supplementary Table S5 online.

*KNN k*-nearest neighbor, *SVM* support vector machine, *MLP* multilayer perceptron, *PPV* positive predictive value, *NPV* negative predictive value, *AUROC* area under the receiver operating characteristic curve.

## Discussion

The first step of this study was to compare the accuracy of dental age estimation by seven DM models with that derived from conventional regression. Both MAE and RMSE were assessed in the internal test sets for both sexes, and the differences between the conventional methods and DM models were 44 ~ 77 days with MAE and 62 ~ 88 days with RMSE. Although the conventional methods were slightly more accurate in this study, it is difficult to conclude whether such a small difference is clinically or practically meaningful. These results imply that dental age estimation by DM models can be performed with almost the same accuracy as the conventional method. Direct comparison with the results of previous studies is difficult because there are no studies comparing the accuracy of DM models with conventional statistical approaches using the same tooth registration technique for the same age span, as was done in this study. Galibourg et al.^24^ compared the MAE and RMSE between two conventional methods (Demirjian’s method^25^ and Willems’ method^29^) and ten DM models in a French population aged 2 to 24 years. They reported that all the DM models proved more accurate than conventional methods, with differences of 0.20 and 0.38 years in MAE, and 0.25 and 0.47 years in RMSE, with Willems and Demirjian’s methods, respectively. Considering the many reports^30–33^ that Demirjian's method inaccurately estimates dental age in populations other than French Canadian, on which the study was based, the difference between the DM models and conventional methods shown in Galibourg's study is quite similar to that observed in this study. Tao et al.^34^ used the MLP algorithm to predict the dental age of 1636 Chinese orthopantomograms, and they also compared the accuracy with the results using Demirjian’s and Willems methods. They reported greater accuracy with MLP than with conventional methods. The differences between DM and conventional methods were < 0.32 years for Demirjian’s method and 0.28 years for Willems’ method, with similar results as this study. The results of these previous studies^24,34^ are also consistent with the results of this study, in that the age estimation accuracy with DM models and conventional methods are similar. However, based on the present results, we can only cautiously conclude that age estimation using the DM model can replace the existing method, as there is currently a lack of comparative and referenceable previous studies. Subsequent studies using more samples are warranted to confirm the results observed in this study.

Among the studies testing the accuracy of dental age estimation with DM, some studies demonstrated higher accuracy than our study. Štepanovský et al.^35^ applied 22 DM models to panoramic radiographs of 976 members of a Czech population aged 2.7 to 20.5 years and verified the accuracy between each model. They evaluated the development of a total of 16 upper and lower left permanent teeth using the classification criteria proposed by Moorrees et al^36^. The MAE was 0.64 to 0.94 years, and the RMSE was 0.85 to 1.27 years, which is more accurate than the two DM models used in this study. Shen et al.^23^ estimated the dental age of seven permanent teeth in the left mandible for Eastern Chinese aged 5 to 13 years using the Cameriere method, which was compared with age estimated by linear regression, SVM, and RF. They showed that all the three DM models had greater accuracy compared to the traditional Cameriere formula. The MAE and RMSE in Shen’s study were lower than those of the DM models in this study. The reason for this increased accuracy in the studies of Štepanovský et al.^35^ and Shen et al.^23^ may be the inclusion of younger subjects in their study samples. Since the age estimation of a participant with developing teeth becomes more accurate as the number of teeth increase during development, when the study participants are younger, the accuracy of the age estimation method derived from it could be higher^37^. In addition, the error in the estimated age with MLP was slightly smaller than that with SLP, which means greater accuracy with MLP than with SLP. MLP was considered slightly more suitable for age estimation, and this may be due to the hidden layer in MLP^38^. However, there is an exception in the case of the female external test set (1.45 for SLP, 1.49 for MLP). Concluding that MLP is more accurate than SLP in estimating age requires more retrospective studies.

The classification performance of the 18-year threshold was also compared between the DM models and conventional methods. All the tested DM models and conventional methods for the internal test sets showed a practically acceptable discrimination level for the 18-year samples. The sensitivity was greater than 87.7% and 94.9%, and the specificity was greater than 89.3% and 84.7% in males and females, respectively. The AUROC was also greater than 0.925 in all the tested models. To the best of our knowledge, there is no study that tested the performance of a DM model for the 18-year classification according to teeth maturity. We can compare the results of this study with the classification performance of deep learning models with panoramic radiographs. Guo et al.^15^ calculated the classification performance for a certain threshold age of a CNN-based deep learning model compared with a manual method based on the Demirjian method. The sensitivity and specificity of the manual method were 87.7% and 95.5%, and those of the CNN model were over 89.2% and 86.6%, respectively. They concluded that a deep learning model could replace or be superior to the manual estimation in the classification of the legal age threshold. The results of this study show similar classification performance; it is thought that classification using the DM models can substitute for age estimation with conventional statistical approaches. Among the DM models, LR was the best model in terms of sensitivity for the male internal test set and with regard to sensitivity and specificity for the female set. LR was the second most accurate in specificity for males. In addition, LR was regarded as one of the more user-friendly DM models^35^ and was less complex and sophisticated in treating data. Based on these results, LR is considered the optimal model for classification performance with the 18-year threshold in the Korean population.

Overall, the accuracy of age estimation or classification performance of the external test set was less accurate or lower compared with results of the internal test set. Several reports indicate that the accuracy or classification performance deteriorates when the age estimation based on Korean population data is applied to the Japanese population^5,39^, and a similar pattern was found in this study. This deterioration tendency was observed in DM models also. Therefore, for accurate age estimation, even when DM is applied in the analysis process, the method derived from own population data should be used as first choice, like the conventional approaches^5,39–42^. Since it is still not clear whether similar tendencies can be shown with deep learning models, a study comparing accuracy and classification performance by applying conventional method, DM models, and deep learning models to the same samples is necessary to confirm whether AI can overcome the limits of ethnic differences in age estimation.

## Conclusions

We confirmed that the conventional method could be replaced by a DM model-based age estimation in forensic practice for age estimation of Koreans. We also found the possibility of introducing ML for forensic age estimation. However, there were also clear limitations, such as an insufficient number of participants in this study to finalize the findings, and a lack of previous studies to compare and verify the results of this study. In future, it will be necessary to conduct DM studies with more samples and in more diverse populations to improve its practical applicability compared to conventional methods. To confirm the possibility of multi-populational use of AI in age estimation, future studies comparing the accuracy and classification performance of DM and deep learning models with conventional methods with the same sample are also needed.

## Materials and methods

### Data collection

This study was conducted with 2657 orthopantomograms, which were collected from Korean and Japanese populations, aged 15 to 23 years. The radiographs from the Koreans were subdivided into a training set of 900 (19.42 ± 2.65 years) and an internal test set of 900 (19.52 ± 2.59 years). The training set was collected from single institution (Seoul St. Mary’s Hospital), and the internal test set was collected from two institutions (Seoul National University Dental Hospital and Yonsei University Dental Hospital). We also collected 857 radiographs from other population data (Iwate Medical University, Japan) for external testing. The radiographs from the Japanese were set as an external test set (19.31 ± 2.60 years). The data were collected retrospectively to analyze the developmental stages of the teeth from panoramic radiographs taken during dental treatment. All the collected data were anonymized, other than sex, date of birth, and the date the radiographs were taken. The inclusion and exclusion criteria were the same as those in previously published studies^4,5^. The chronological age of the samples was calculated by subtracting the date of birth from the date the radiographs were taken. The sample group was classified into nine age groups. The age and sex distribution are shown in Table 3. This study was conducted in compliance with the Declaration of Helsinki and approved by the Institutional Review Board (IRB) of Seoul St. Mary’s Hospital, the Catholic University of Korea (KC22WISI0328). Since this study has a retrospective design, it is impossible to obtain informed consent from all patients who had radiographs for treatment purposes. The requirement for informed consent was waived by the IRB of Seoul St. Mary’s Hospital, the Catholic University of Korea.

**Table 3:** Age and sex distribution of the samples.

Age group (years)	Training set			Internal test set			External test set		
	Male	Female	Total	Male	Female	Total	Male	Female	Total
15	50	50	100	50	50	100	48	65	113
16	50	50	100	50	50	100	44	61	105
17	50	50	100	50	50	100	42	47	89
18	50	50	100	50	50	100	45	57	102
19	50	50	100	50	50	100	47	47	94
20	50	50	100	50	50	100	46	43	89
21	50	50	100	50	50	100	45	45	90
22	50	50	100	50	50	100	45	43	88
23	50	50	100	50	50	100	44	43	87
Total	450	450	900	450	450	900	406	451	857

### Evaluation of dental maturity

The developmental stages of the second and third molars of both jaws were evaluated according to Demirjian’s criteria^25^. If the same type of teeth were found on the right and left sides of each jaw, only one tooth was selected. If the homologous teeth on each side were at different stages of development, the tooth with the lower stage of development was selected to consider the uncertainty of the estimated age^4^. One hundred randomly selected radiographs from the training set were evaluated by two experienced observers to test inter-observer reliability after pre-calibration for staging of dental maturity. Intra-observer reliability was tested with two times-evaluation by the main observer at an interval of three months.

#### Regression and classification with DM models

The sex and developmental stages of the second and third molars of each jaw of the training set, which were evaluated by the main observers, were trained by various DM models and the chronological ages were set as target values. The SLP and MLP models, widely used for the machine learning, were tested for the regression algorithms. The DM models used the developmental stages of four teeth for the combination of linear functions and converged these data to estimate age. An SLP is the simplest neural network and does not contain any hidden layer. The working SLP is based on the threshold transfer between the nodes. The SLP model in regression is mathematically the same as multivariable linear regression. The MLP model has more than one hidden layer with non-linear activation functions, unlike the SLP model. We used one hidden layer for our experiment, which only had 20 hidden nodes with non-linear activation functions. Gradient descent was used as the optimization method, and the MAE and RMSE were used as loss functions to train our machine learning models. The obtained best regression model was applied to the internal and external test sets, and the dental ages were estimated.

Classification algorithms were developed to predict whether the age of the sample reached the age of 18 years using the maturity of four teeth on the training set. To build the models, we derived seven representation machine learning algorithms^6,43^: (1) LR, (2) KNN, (3) SVM, (4) DT, (5) RF, (6) XGBoost, and (7) MLP. LR is one of the most widely used classification algorithms^44^. It is a supervised learning algorithm that uses regression to predict the probability that data fall into a category from 0 to 1 and classify the data as belonging to a more likely category according to that probability; it is mainly used for binary classification. KNN is one of the simplest machine learning algorithms^45^, which, when given new input data, finds k data close to the existing set and then classifies it as the class with the highest frequency of occurrence. We set three as the number of neighbors to consider (*k*). SVM is an algorithm that maximizes the distance between two classes by extending a linear space into a nonlinear space using a kernel function, and the distance is called a margin^46^. For this model we used bias = 1, power = 1, and gamma = 1 as the hyperparameters of polynomial kernels. The DT is used in various fields as an algorithm that classifies the entire data set into several sub-groups by representing decision rules in a tree structure^47^. The model was set to two as the minimum number of records per node, and the Gini index was used as the quality measure. RF is an ensemble technique that combines multiple DTs to improve performance using a bootstrap aggregating technique, which generates a weak classifier for each sample by randomly extracting samples of the same size multiple times from the original data set^48^. We used 100 trees, 10 tree depth, 1 minimum node size, and the Gini impurity index as a node splitting criterion. The classification of new data was determined by a majority vote. XGBoost is an algorithm of the ensemble's boosting technique and uses a method of inputting errors between actual and predicted values from previous models as training data, and supplementing errors using gradients^49^. It is a widely used algorithm because of good performance and resource efficiency and is characterized by its strong durability as an overfitting regulatory function. The model was set to 400 boosting rounds. MLP is a neural network in which one or more perceptrons form multiple layers, with one or more hidden layers between the input and output layers^38^. Using this, nonlinear classification is possible, and when an input layer is put in and a result value comes out, the result value of the prediction and the actual result value are compared, and the error is backpropagated. We set one hidden layer and 20 hidden neurons per layer. Each model that we developed was applied to the internal and external sets to test classification performance by calculating the sensitivity, specificity, PPV, NPV, and AUROC. Sensitivity was defined as the ratio of samples that reached the age of 18 years and were estimated as equal or over the age of 18 years. Specificity was the ratio of samples that were under the age of 18 years and were estimated as under the age of 18 years.

### Regression with conventional statistics

The evaluated teeth stages in the training set were converted to numeric stages for statistical analysis. Multivariable linear and logistic regression were performed to develop a predictable model for each sex, and the regression formulae, which can be used in age estimation, were derived. We estimated the dental ages of the internal and external test sets with these formulae. Table 4 shows the regression and classification models used in this study.

**Table 4:** Regression and classification models used in this study.

Method	Regression model	Classification model
Conventional	Multivariable linear regression	Multivariable linear regression
		Multivariable logistic regression
Data mining	Single layer perceptron	K-nearest neighbor
	Multilayer perceptron	Support vector machine
		Logistic regression
		Decision tree
		Random forest
		XGBoost
		Multilayer perceptron

The regression model was used to calculate the accuracy of the estimated age, and the classification model was used to classify performance for the internal and external test sets.

### Statistical analysis

Intra- and inter-observer reliabilities were calculated using Cohen’s kappa statistics. To test the accuracy of the DM and conventional regression models, we calculated the MAE and RMSE with the estimated and chronological ages of the internal and external test sets. These errors are often used to evaluate the accuracy of model predictions, and the smaller the error, the higher the accuracy of the prediction^24^. The MAE and RMSE of the internal and external test sets, calculated with both DM and conventional regression, were compared. The classification performance of the 18-year threshold in conventional statistics was evaluated with a two-by-two contingency table. The calculated sensitivity, specificity, PPV, NPV, and AUROC for the test sets were compared with the measures for the DM classification models. Data are expressed as means ± standard deviations, or number (%), based on the characteristics of the data. A two-tailed P-value < 0.05 was considered statistically significant. All conventional statistical analyses were performed using SAS version 9.4 (SAS Institute, Cary, NC). The DM regression models were implemented in a Python with Keras^50^ 2.2.4 with Tensorflow^51^ 1.8.0 backend dedicated to mathematical operation. DM classification models were implemented in the Waikato environment for knowledge analysis, and the Konstanz Information Miner (KNIME) analytics platform 4.6.1^52^.

## Supplementary Information

Supplementary Information.

## Data Availability

The authors confirm that the data supporting the findings of this study are available within the article and its Supplementary material. The datasets generated and/or analyzed during the study can be available from the corresponding author upon reasonable request.
