# Supplementary material for: Validation of data mining models by comparing with conventional methods for dental age estimation in Korean juveniles and young adults
Source: Sci Rep. 2023 Jan 13;13:726. doi: 10.1038/s41598-023-28086-1 (PMC9839668; doi:10.1038/s41598-023-28086-1)
Supplement: Supplementary file 1 — Supplementary Information. [file 41598_2023_28086_MOESM1_ESM.pdf]

Validation of data mining models by comparing with conventional methods for dental age estimation in Korean juveniles and young adults

Akiko Kumagai[1], Seoi Jeong[2], Daeyoun Kim[3], Hyoun-Joong Kong[4,5,6], Sehyun Oh[7], and Sang-Seob Lee[7,*]

[1]Division of Forensic Odontology and Disaster Oral Medicine, Department of Forensic Science, Iwate Medical University, Iwate, 028-3694, Japan

[2]Interdisciplinary Program in Bioengineering, Graduate School, Seoul National University, Seoul, 03080, Republic of Korea

[3]Kakao Corp., Jeju, 63309, Republic of Korea

[4]Transdisciplinary Department of Medicine and Advanced Technology, Seoul National University Hospital, Seoul, 03080, Republic of Korea

[5]Medical Big Data Research Center, Seoul National University College of Medicine, Seoul, 03080, Republic of Korea

[6]Department of Biomedical Engineering, Seoul National University College of Medicine, Seoul, 03080, Republic of Korea

[7]Department of Anatomy · Catholic Institute of Applied Anatomy, College of Medicine, The Catholic University of Korea, Seoul, 06591, Republic of Korea

[*]sslee1418@gmail.com

		Training set					
		Male			Female		
Tooth	Stage	n	Mean	SD	n	Mean	SD
UM2	A						
	B						
	C						
	D						
	E						
	F				1	15.01	0.00
	G	135	16.14	0.96	151	16.62	1.07
	H	315	20.77	1.88	298	20.91	1.83
UM3	A						
	B						
	C	5	15.37	0.47	4	15.39	0.26
	D	38	15.44	0.53	39	16.08	1.00
	E	73	16.70	1.26	100	17.32	1.51
	F	86	18.28	1.75	122	19.01	1.77
	G	78	19.66	1.51	77	20.68	1.59
	H	170	21.97	1.23	108	22.44	1.07
LM2	A						
	B						
	C						
	D						
	E						
	F	1	15.27	0.00	1	15.01	0.00
	G	129	16.25	1.07	161	16.66	1.03
	H	320	20.66	2.03	288	21.04	1.73
LM3	A						
	B						
	C	11	15.32	0.41	6	15.85	0.66
	D	29	15.36	0.54	53	16.33	1.32
	E	58	16.48	1.36	75	17.45	1.73
	F	99	17.83	1.29	105	18.60	1.68
	G	90	19.77	1.25	122	20.69	1.58
	H	163	22.14	1.14	89	22.58	0.99

Supplementary Table S1. Mean and standard deviation of chronological age by Demirjian's stage of second and third molars in the training set. *SD* standard deviation, *UM2* maxillary second molar, *UM3* maxillary third molar, *LM2* mandibular second molar, *LM3* mandibular third molar.

		Internal test set					
		Male			Female		
Tooth	Stage	n	Mean	SD	n	Mean	SD
UM2	A						
	B						
	C						
	D						
	E						
	F				1	15.88	0.00
	G	155	16.70	1.15	125	16.40	0.89
	H	295	21.00	1.78	324	20.73	1.95
UM3	A						
	B	1	16.89	0.00			
	C	1	16.79	0.00	6	15.64	0.50
	D	22	16.11	1.13	33	16.08	1.04
	E	76	17.01	1.54	103	17.13	1.38
	F	114	18.27	1.71	147	19.46	1.57
	G	78	19.55	1.62	59	20.66	1.70
	H	158	22.12	1.36	102	22.68	1.01
LM2	A						
	B						
	C						
	D						
	E						
	F	3	15.45	0.25	3	15.33	0.48
	G	162	16.84	1.20	143	16.56	0.89
	H	285	21.08	1.76	304	20.95	1.80
LM3	A						
	B				2	15.46	0.27
	C	4	15.35	0.48	6	16.46	1.10
	D	28	16.33	1.31	61	17.74	1.68
	E	56	16.21	1.16	75	19.11	1.64
	F	115	16.94	1.53	137	21.27	1.64
	G	126	17.98	1.51	100	22.89	0.89
	H	121	20.29	1.82	69	15.46	0.27

Supplementary Table S2. Mean and standard deviation of chronological age by Demirjian's stage of second and third molars in internal test set. *SD* standard deviation, *UM2* maxillary second molar, *UM3* maxillary third molar, *LM2* mandibular second molar, *LM3* mandibular third molar.

		External test set					
		Male			Female		
Tooth	Stage	n	Mean	SD	n	Mean	SD
UM2	A						
	B						
	C						
	D	1	16.08	0.00			
	E						
	F	8	16.19	1.23	7	16.41	1.26
	G	97	16.76	1.51	139	17.39	1.98
	H	300	20.46	2.17	305	20.03	2.41
UM3	A						
	B				2	15.88	0.79
	C	4	15.94	0.81	10	16.07	0.97
	D	21	15.94	0.76	53	17.03	1.91
	E	54	16.90	1.53	79	17.07	1.60
	F	69	17.52	1.65	89	18.60	1.99
	G	102	19.67	1.72	113	19.96	1.93
	H	156	21.69	1.63	105	21.80	1.79
LM2	A						
	B						
	C						
	D						
	E						
	F	2	15.57	0.30	5	16.03	0.56
	G	110	17.12	1.86	128	17.27	1.92
	H	294	20.39	2.24	318	19.97	2.42
LM3	A						
	B	1	15.04	0.00	3	15.12	0.14
	C	6	15.87	0.56	29	16.50	1.21
	D	28	16.17	0.93	65	16.83	1.55
	E	50	16.35	1.48	71	17.71	1.79
	F	65	17.77	1.57	66	18.24	1.85
	G	119	19.81	1.68	130	20.30	1.85
	H	137	21.90	1.47	87	22.13	1.47

Supplementary Table S3. Mean and standard deviation of chronological age by Demirjian's stage of second and third molars in external test set. *SD* standard deviation, *UM2* maxillary second molar, *UM3* maxillary third molar, *LM2* mandibular second molar, *LM3* mandibular third molar.

	Intra-observer reliability			Inter-observer reliability		
	Cohen's kappa	95% CI	P-value	Cohen's kappa	95% CI	P-value
UM2	0.940	0.873-1.000	<.001	0.900	0.815-0.985	<.001
UM3	0.908	0.842-0.974	<.001	0.856	0.776-0.935	<.001
LM2	0.940	0.873-1.000	<.001	0.900	0.814-0.985	<.001
LM3	0.934	0.878-0.990	<.001	0.921	0.859-0.982	<.001
Total	0.941	0.914-0.967	<.001	0.911	0.879-0.943	<.001

Supplementary Table S4. Intra- and inter-observer reliability for the evaluation of maturity of second and third molars in the training set. *CI* confidence interval, *UM2* maxillary second molar, *UM3* maxillary third molar, *LM2* mandibular second molar, *LM3* mandibular third molar.

Training set	Sensitivity	Specificity	PPV	NPV	AUROC
Male	0.958<br>(0.928-0.977)	0.951<br>(0.902-0.980)	0.977<br>(0.953-0.991)	0.913<br>(0.856-0.953)	0.995<br>(0.989-1.000)
Female	0.964<br>(0.937-0.982)	0.986<br>(0.950-0.998)	0.993<br>(0.976-0.999)	0.927<br>(0.873-0.963)	0.995<br>(0.990-1.000)

Internal test set	Sensitivity	Specificity	PPV	NPV	AUROC
Male	0.974<br>(0.949-0.989)	0.953<br>(0.906-0.981)	0.977<br>(0.953-0.991)	0.947<br>(0.898-0.977)	0.990<br>(0.982-0.997)
Female	0.949<br>(0.918-0.971)	0.978<br>(0.937-0.996)	0.990<br>(0.971-0.998)	0.893<br>(0.833-0.938)	0.998<br>(0.996-1.000)

External test set	Sensitivity	Specificity	PPV	NPV	AUROC
Male	0.846<br>(0.801-0.885)	0.870<br>(0.788-0.929)	0.952<br>(0.920-0.974)	0.649<br>(0.562-0.730)	0.912<br>(0.881-0.942)
Female	0.797<br>(0.748-0.841)	0.766<br>(0.688-0.832)	0.878<br>(0.833-0.914)	0.642<br>(0.565-0.713)	0.873<br>(0.841-0.905)

Supplementary Table S5. The classification performance with 95% confidence interval of the conventional method based on the 18-year threshold. AUROC was obtained by multivariable logistic regression analysis, and the others were calculated using multivariable linear regression anaylsis. The 95% confidence interval did not apply in the data mining classification model. *PPV* positive predictive value, *NPV* negative predictive value, *AUROC* area under the receiver operating characteristic curve.

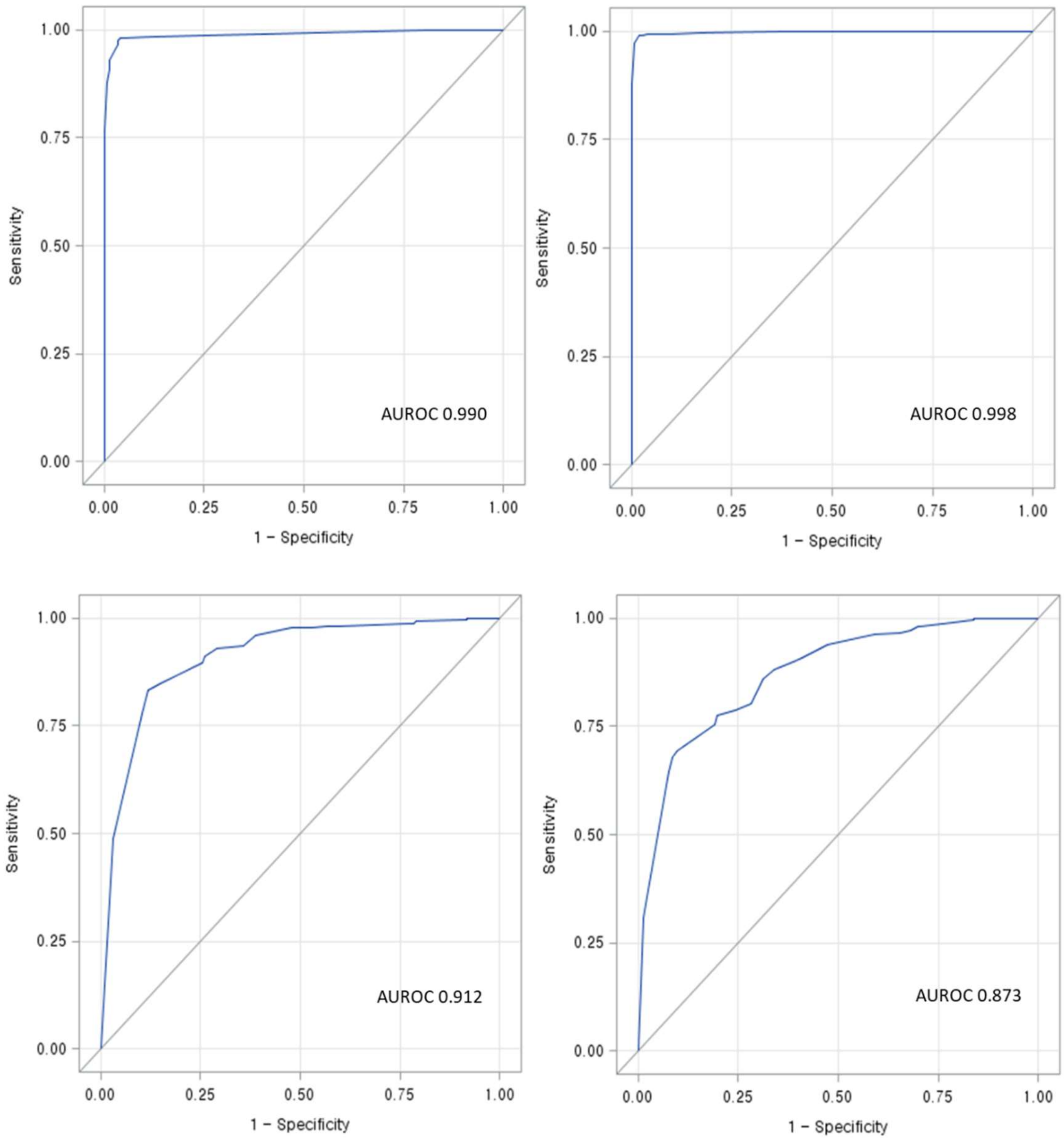

Supplementary Figure S1. ROC curves for conventional methods in internal (top) and external (bottom) test sets. AUROC was obtained by multivariable logistic regression analysis and was 0.990 for male and 0.998 for female. *ROC* receiver operating characteristic, *AUROC* area under the receiver operating characteristic curve. (left: male, right: female)
